# Use of Supplemented or Human Material to Simulate PD Behavior of Antibiotics at the Target Site In Vitro

**DOI:** 10.3390/pharmaceutics12080773

**Published:** 2020-08-14

**Authors:** Alina Nussbaumer-Pröll, Markus Zeitlinger

**Affiliations:** Department of Clinical Pharmacology, Medical University of Vienna, 1090 Vienna, Austria; alina.nussbaumer-proell@meduniwien.ac.at

**Keywords:** adapted growth media, body fluids, in vitro, PD, MIC, TKC, MHB

## Abstract

In antimicrobial drug development, in vitro antibiotic susceptibility testing is conducted in standard growth media, such as Mueller–Hinton broth (MHB). These growth media provide optimal bacterial growth, but do not consider certain host factors that would be necessary to mimic the in vivo bacterial environment in the human body. The present review aimed to include relevant data published between 1986 and 2019. A database search (PubMed) was done with text keywords, such as “MIC” (minimal inhibitory concentration), “TKC” (time kill curve), “blood”, “body fluid”, “PD” (pharmacodynamic), and “in vitro”, and 53 papers were ultimately selected. Additionally, a literature search for physiologic characteristics of body fluids was conducted. This review gives an excerpt of the complexity of human compartments with their physiologic composition. Furthermore, we present an update of currently available in vitro models operated either with adapted growth media or body fluids themselves. Moreover, the feasibility of testing the activity of antimicrobials in such settings is discussed, and pro and cons for standard practice methods are given. The impact on bacterial killing varies between individual adapted microbiological media, as well as direct pharmacodynamic simulations in body fluids, between bacterial strains, antimicrobial agents, and the compositions of the adjuvants or the biological fluid itself.

## 1. Introduction

To predict in vivo efficacy of antimicrobials in humans, different strategies have been pursued. Over the past decades, there has been lively debate regarding whether in vitro models (e.g., time kill curves (TKCs), minimal inhibitory concentration (MIC), or dynamic in vitro models) or animal models (e.g., mouse thigh infection models, skin and soft tissue models, urinary tract infection (UTI) models), are more suitable for the linking of preclinical data to in vivo efficacy in patients [1]. Animal models, when carefully controlled, are a very powerful tool, as they can provide accurate and predictive data [2]. Animal testing, however, is currently facing unprecedented levels of criticism. Additionally, the use of in vitro models for the simulation of human pharmacokinetic (PK)/ pharmacodynamic (PD) profiles might be more cost-effective and convenient than the use of animal models [1,3]. Hence, in vitro antibiotic susceptibility testing provides the basis to describe epidemiological changes in bacterial populations, and is frequently used to estimate clinical breakpoints.

These in vitro tests have in common that they are commonly conducted in pure Mueller–Hinton broth (MHB), a growth medium that is ideal for bacterial growth, but does not resemble an environment representative of the in vivo situation. The lack of cellular interactions, host–bacteria interactions, and relevant physiological proteins or other components in culture media are important factors to consider. Thus, different attempts have been taken to overcome these limitations specific to in vitro experiments. On the one hand, different additives of human origin (e.g., defibrinated blood, serum, or albumin) to MHB have been used for antimicrobial susceptibility testing, but studies have also been conducted in human body fluids (e.g., urine, cerebrospinal fluid (CSF), bile). Moreover, the impact of different standard culture media on antibiotic activity has been investigated. A study by Kumaraswamy et al. revealed that azithromycin lacks activity against *Stenotrophomonas maltophilia* in standard MIC testing, but synergizes with cationic peptide antibiotics to kill these Gram-negative bacilli in a medium mimicking tissue fluid conditions [4]. Even though the impact of different standard growth media on antibiotic susceptibility testing might be an important factor to consider, we focused on the following approaches.

In this review, we aim to give an excerpt of the complexity of human compartments with their physiologic composition, as it is important to understand the actual environment of the bacterial infection site. Further, we present an update of currently available in vitro models, operated either with adapted growth media or body fluids themselves.

Thus, we give an overview of already performed studies and point out which areas are well explored, which rather need improvement or even have been untouched up to know. Moreover, we discuss the feasibility of testing the activity of antimicrobials in such settings, and give pros and cons for standard practice methods.

## 2. Complexity of Human Compartments and Their Physiologic Composition

The human body is inherently dynamic, and drug concentrations within it change due to metabolism and diffusion into the tissue. This, in turn, can change the clinical efficacy of antibiotics against bacteria. Some drugs penetrate certain tissues and body fluids better than others. pharmacokinetic (PK)/pharmacodynamic (PD) indices play an important role in antimicrobial drug development and post-marketing dose optimization, as they contribute to the optimization of the effects of antibiotics in vivo. PK parameters describe the presence of the drug in plasma or tissue fluids in the body over time, including its absorption, distribution, metabolism, and elimination. PD parameters describe the interaction between the concentration of the drug at the target site and the physiological response [3]. These PK/PD indices vary within different human compartments, and might be influenced by the specific composition of the target site. Thus, it is important to consider the physiologic composition of bacterial infection sites when conducting in vitro experiments.

The physiological composition of the most common compartments for bacterial infection, such as blood, urine, brain/spinal cord, and the lung, is shown in Table 1. This excerpt of the human body shows how different the target body fluids are composed.

Blood, for example, consists of 45% hematocrit, harboring different corpuscular blood components as erythrocytes, leukocytes, and platelets. The other 55% are made up by plasma, which consists of around 90% of water and 10% of soluble proteins [5]. In some aspects, pure MHB closely resembles human plasma and serum (content of Na^+^, K^+^, and Cl^−^, as well as similarities within pH and osmolality) [6], but MHB lacks corpuscular blood components or other cells, and the impact on antibiotic activity in in vitro experiments might be falsified compared to the in vivo situation (e.g., less antibiotic activity against pathogens in the presence of corpuscular blood components due to binding, or diffusion of the antibiotic into the cells). Moreover, the composition of human urine, cerebrospinal fluid (CSF), epithelial lining fluid (ELF), bile, pancreatic juice, or even breast milk vary with regard to pH, protein content, sugar content, vitamins, ions, and immune globulins—i.e., heterogenic conditions that cannot be achieved by one single growth media.

## 3. Published In Vitro Studies

In the previous section, we depicted the physiologic composition of bacterial infection sites. In this section, we will summarize already published in vitro studies, with the intention to test in adapted standard growth media (Table 2) or in human body fluids (Table 3). Additionally, an excerpt of the impact of these adaptions in standard susceptibility testing on antibiotic activity is depicted for each study in the Supplementary Materials (Table S1).

### 3.1. Adapted Growth Media

In this part of the review, an extensive literature search has been done on studies that have spiked MHB with different body fluids or blood components.

One very important advantage of testing with adapted MHB is the necessary volume used in the experiments. Compared to experiments conducted solely in body fluids, less of the human material is needed, as MHB can be used as the carrier fluid. Nevertheless, MHB might not always represent an ideal media to ensure the integrity of the additives compared to the body fluid itself. However, these publications show that data generated by experiments conducted in pure MHB compared to adapted MHB might show inconsistent results (e.g., an elevated MIC is found in the adapted setting compared to the MHB setting). In the following paragraphs, studies that have investigated the impact of different adjuvants (e.g., albumin, plasma, or corpuscular blood components) compared to growth media are discussed and have been further summarized above in Table 2.

As already shown in Table 1 above, blood is a very complex entity. Thus, various studies have investigated different components of this body fluid, which makes up around 7% of a human’s body weight.

Even though only 10% of blood plasma is made up soluble substances, of which 70% are plasma proteins, protein binding (PB) is an intensely discussed topic, with a huge effect on in vitro testing and in vivo efficacy.

A review discussing this topic has revealed that conducting antibiotic susceptibility testing in protein-free standard medium versus a protein-rich medium may lead to flawed conclusions, as antibiotic activity might be present in vitro but not in vivo [34].

Furthermore, the influence of PB on antimicrobial activity has been reported in numerous publications, yet there is no standardized method for pharmacodynamic models in in vitro testing that takes PB in account [18,19,34,35].

Different factors that impact the outcome of such experimental settings, including the source of protein (e.g., bovine, pig, or human), the type of processing (e.g., freshly collected plasma, blood bank processed, or bio-repository purchased), the concentration of the tested antibiotic, temperature, pH, electrolytes, and supplements have been described as impacting the viability of the biological matrix and the subsequent degree of PB [34]. To be more specific, most studies testing the impact of PB on antibiotic activity have used MHB as a protein-free reference media that closely resembles human plasma and serum (content of Na^+^, K^+^, Cl^−^, and similarities in pH and osmolality) [6]. To investigate the impact of PB, MHB is then spiked with serum or albumin [19,21,36]. Even though 100% serum might be a good option to resemble in vivo conditions, studies have already shown that pure serum is less optimal in terms of bacterial growth compared to standard growth media, such as MHB [19,37]. Thus, many studies test the impact of the addition of serum to growth media in various concentrations, ranging from 20% to 100% [16,38,39,40,41,42,43,44,45].

Human albumin has also been considered as a protein supplement and an alternative to serum. However, this has faced the major limitation that non-albumin proteins that might be involved in PB are neglected [21,46]. Thus, the addition of albumin might not provide the same PB capacity as found in other experimental approaches using human serum [18,19].

Further blood components, such as plasma, have been investigated as well. One study showed that in MHB and MHB spiked with plasma, the antimicrobial activity of fusidic acid was affected by the presence of human plasma (MIC elevations of 4-fold to >128-fold) [20].

In vitro antibiotic susceptibility testing in MHB with the addition of 65% defibrinated human blood against various bacterial strains, using different antimicrobials, overall showed a negative impact on antibiotic activity in the adapted setting [22,23,47,48,49,50].

Finally, two studies have also investigated the impact of corpuscular blood components (erythrocytes and platelets) in MHB on antibiotic activity against Gram-positive and Gram-negative American Type Culture Collection (ATCC) strains. They showed that erythrocytes did not impact bacterial growth of tested bacterial strains, but lowered the antibiotic activity of meropenem, ciprofloxacin, and tigecycline to a different extent depending on the pathogen tested [14]. In the platelet setting, these studies showed that no impact on bacterial growth was present, but pathogens affect platelet concentrations depending on the bacterial strain. The impact of platelets on antibiotic stability and activity was present in certain cases, but this varied between different individual thrombocyte concentrates [15].

Contrary to blood, urine harbors only a negligible number of cells. However, pH might be the important factor to consider in the following in vitro experiment, as urinary pH is, amongst other factors, dependent on the diet, and might range from 5.0 to 8.0 [51].

A study conducted with MHB spiked with 90%, 80%, and 50% human urine against 16 uropathogens showed that the presence of an acidic pH (pH 5.0–6.0) led to enhanced activity of delafloxacin against the tested strains [24].

Furthermore, even the addition of ions (Ca^2+^ and Mg^2+^) to MHB might impact antibiotic activity. Different cation concentrations in MHB were tested on the antimicrobial activity of colistin by both broth microdilution and time kill studies. The authors could reveal that MIC values of colistin against the majority of isolates of both *P. aeruginosa* and *Acinetobacter baumannii* (*A. baumannii*) increased significantly with higher cation concentrations, and the MICs of *E. coli* decreased with ascending cation concentrations [52]. Furthermore, supplementation of broth with various cation ions has been discussed—e.g., calcium supplementation was shown to be beneficial for daptomycin activity [53], iron depleted media was required for the accurate depiction of cefiderocol activity [54], and meropenem in vivo efficacy was best represented by the pharmacodynamic profile generated using MICs determined in zinc-depleted media against Metallo-ß-lactamase-resistant *Enterobacteriaceae* both in vitro and in vivo [55].

These studies show that antibiotic activity can already be impacted by pH or ions, which are factors that are compared to others—which is more easily achieved within in vitro testing.

A further approach to better reflect the in vivo activity in vitro is the addition of surfactant to MHB. Pulmonary surfactant is a surface-active lipoprotein complex (phospholipoprotein), and might be an important factor to consider. A study tested in vitro antimicrobial activity of amoxicillin against *S. aureus* and *Streptococcus pneumoniae* (*S. pneumoniae*), as well as ceftazidime and tobramycin against *Klebsiella pneumoniae* (*K. pneumoniae*), *P. aeruginosa*, *S. aureus*, and *S. pneumoniae* in the presence or absence of surfactant (isolated from bovine lungs). They concluded that both amoxicillin and ceftazidime can be combined with surfactant without a loss of activity. On the contrary, higher concentrations of tobramycin were required for bacterial killing in the presence of surfactant when compared to MHB [56]. In another study, the authors were able to confirm reduced daptomycin activity against *S. aureus* ATCC-29213 when experiments were conducted with a surfactant (Survanta) compared to MHB [57]. Indeed, another publication verifies the findings of prior studies regarding reduced daptomycin activity in the presence of surfactant (porcine surfactant; Curosurf) against *S. aureus* ATCC-29213. In addition, the authors could show that the antibiotics linezolid, doripenem, tigecycline, and moxifloxacin also showed reduced antibiotic activity against the tested strains [58].

In contrast, other studies with telavancin and an expanded-spectrum lipopeptide were not affected in their activity with in vitro testing with the presence of surfactant (Survanta) [59,60].

Thus, to sum up, in vitro studies with defibrinated blood, plasma, serum, albumin, and corpuscular blood components simulating the environment of human blood have been conducted. Furthermore, compartments like the lung or the urinary tract have been simulated with the addition of surfactant or urine, respectively. In Table 2, these publications are presented with summaries of the test methods (MIC, TKC, etc.), bacterial strains, and the antibiotics involved.

The diversity of studies emphasizes that even though testing in adapted media is considered important for antibiotic susceptibility testing, no standardized methods have evolved up to now.

### 3.2. Body Fluids as Growth Media

Contrary to adapted media, antibiotic susceptibility testing in human body fluids faces different problems, as obtaining potential media is sometimes difficult, the extracted volume is often not enough to test in multiple replicates, or indeed the process of extraction of the body fluid itself is not feasible in human subjects. Therefore, these kinds of studies are sometimes limited based on the practicability of their source.

Nevertheless, studies of antibiotic susceptibility testing conducted in human body fluids has revealed noteworthy results.

Urine is one of the more easily obtained body fluids, and has been used for antibiotic susceptibility testing in various studies.

Zeiler et al. showed that an acidic environment in pooled human urine negatively impacted the antibacterial activity of ciprofloxacin, norfloxacin, and ofloxacin against *E. coli*, *S. aureus*, and *P. aeruginosa* compared to standard growth media with a neutral pH [61]. Furthermore, another study observed a reduction in the activity of fluoroquinolones, such as ciprofloxacin, levofloxacin, and moxifloxacin against *E. coli* ATCC-25922 and *Klebsiella oxycota* (*K. oxycota*) ATCC-29213 in pooled human urine and MHB, confirming previous observations from older compounds [31]. Moreover, a comparable setting testing the activity of trimethoprim, fosfomycin, amikacin, colistin, and ertapenem in human urine against *E. coli* ATCC-25922, *K. oxycota* ATCC-29213, *Proteus mirabilis* (*P. mirabilis*) ATCC-14153, and *Enterococcus faecalis* (*E. faecalis*) ATCC-29212 revealed once again reduced antibiotic activity in settings below pH 6 [29].

Furthermore, pH-dependent, reduced antibiotic activity was found for 18 out of 24 widely used antibiotics, ranging from fluoroquinolones to beta-lactams, revealing that many, but not all, antibiotics show less activity in acidic pH, as summarized in the study by Yang et al. [28].

Cerebrospinal fluid (CSF) might also act as an important environment for antibiotic activity, especially in the treatment of central nervous infections, such as meningitis or ventriculitis. Concerns over equality of the antibiotic activity in vitro and its clinical efficacy have been discussed in the review by Matzneller et al. [11]. Indeed, one study showed that rifampicin concentrations above the MIC were required to achieve bacterial killing of *S. aureus* in CSF (with CO_2_) compared to pure MHB. In contrast, bacterial eradication could be achieved with lower concentrations of cefepime compared to MHB in CSF (with CO_2_) [33]. Another study also indicated enhanced activity of linezolid against *S. aureus* in CSF [30]. However, a further publication showed impaired antibiotic activity of fosfomycin in in vitro tests with CSF [32].

Moreover, a study investigated the impact of human bile on bacterial killing of *E. faecalis* ATCC-29212 and *E. coli* ATCC-25922. In all experiments, the presence of bile reduced the antibiotic activity of linezolid, tigecycline, ciprofloxacin, and meropenem against the test strains [26]. To conclude, testing in human body fluids enables the comparison of results of current antimicrobial susceptibility testing, and is compiled in Table 3.

Additionally, in the Supplementary Materials (Table S1), the impact of the tested adjuvants and the body fluids on antibiotic activity is rated as negative (↓: decrease in antibiotic activity), positive impact (↑: increase in antibiotic activity), or no difference between adjusted and pure growth media (↔: no impact on antibiotic activity).

Generally, a negative impact on antibiotic activity was observed for the different PB settings conducted with albumin, serum, and plasma compared to pure MHB. Furthermore, most studies with pooled human urine set to an acidic pH did negatively impact the activity of the tested antimicrobials compared to MHB. While the addition of surfactant to MHB did also reveal a reduced activity of certain antibiotics against the tested strains, activity of most antibiotics tested in MHB spiked with defibrinated blood compared to MHB revealed an increase in antibiotic activity.

Overall, Table S1 (Supplementary Materials) reveals that there is a trend of a reduced activity of antimicrobials tested in adapted media or body fluids compared to pure MHB, but depicts as well that the degree of the impact varies between bacterial strains, antimicrobial agents, and the compositions of the adjuvants or the biological fluid itself.

## 4. Standard Practice Methods: Pros and Con for Adapted Media

The in vitro studies listed above mainly use MIC and TKC methods for antibiotic susceptibility testing. Besides disc-diffusion tests, these methods are the most important tools for antimicrobial susceptibility testing. Thus, in the following the pros and con for these methods is briefly described, and are additionally summarized in Table 4 below.

### 4.1. MIC and MBC

The MIC is defined by the lowest antibiotic concentration needed to suppress visible growth of a pathogen after 18–20 h incubation at 37 °C, with an initial inoculum of 5 × 10^5^ colony forming units (CFU)/mL [12]. The dose of most antibiotics is adjusted to obtain unbound plasma concentrations in some relation to the MIC for a respective pathogen; these relationships are known as PK/PD parameters of time- and concentration-dependent antibiotics [13]. An elegant way to describe the MIC is the equation explained by Mouton and Vink [62,63] and Schuck [64], in which the different factors influencing the numerical value of a MIC are formally explained.

(1) $$MIC=EC_{50}\times {\left(\frac{K_{Growth}-0.29}{Emax-\left(K_{Growth}-0.29\right)}\right)}^{\frac{1}{Gamma}}$$

For all antimicrobial drugs of all antibiotic classes, the PD profile of, i.e., the concentration–effect relationship, can be described by three fundamental PD parameters. The efficacy (*Emax*, typically the maximal killing rate as obtained in TKC), the potency (*EC*_50_, i.e., the antibiotic concentration to achieve half-the killing rate), and the sensitivity (*Gamma* reflects the slope more or less of the concentration–effect relationship, which is characteristic for the different classes of antibiotics). These three fundamental properties have a pharmacological meaning, and are of interest when investigating some pathophysiological mechanisms (including the development of resistance) especially, when one wants to extrapolate data of in vitro tests to in vivo efficacy.

In addition, this equation formally includes test tube conditions that are specific to the conditions of having MIC testing as the net growth rate (*K_Growth_*, which is actually *K_Growth_* - *K_Death_*), the time of measurement (growth = 18–24 h), and the initial bacterial load, e.g., 5 × 10^5^ CFU/mL; these are depicted in the formula by *K_Growth_* = −0.29, and is explained in more detail by Mouton and Vink [63].

Even though the MIC is seen as the most important PD value and is routinely tested in microbiology (used in various studies listed above), antibiotic activity is a dynamic process, and the MIC is only a one-point measurement, creating a black and white image. Small changes in the anti-infective activity are neglected, as concentrations near the MIC will still show antimicrobial activity compared to concentrations close to zero [13]. Another limitation of the determination of the MIC might be turbidity of certain growth media or adjuvants, which complicates the accurate evaluation of the MIC and might limit experimental settings. Indeed, this might be the biggest challenge when testing with adapted MHB (e.g., with blood, albumin, surfactants) or directly with body fluids (e.g., mother’s milk, ascites). Furthermore, bacteria with specific characteristics—for example, swarming or CO_2_ production, might lead to contamination of wells, and therefore might falsify the results.

The determination of the minimal bactericidal concentration (MBC) is also a valid in vitro method. The MBC is defined as the lowest concentration of an antibiotic that achieves ≥99.9% killing of the initial bacterial inoculum in a time frame of 24 h [15]. The determination is done by plating aliquots drawn from each broth dilution on agar plates. The MBC is not dependent on a visual evaluation, as is the case with the MIC, and factors such as turbidity may not impact the results, and thus might be favorable when testing with adapted MHB settings. Furthermore, the MBC is complementary to the MIC, as the concentration determined as the MIC might show no visual growth, but by plating an aliquot of these bacteria onto agar, CFUs might still be detected.

Although we must consider that the MIC and the MBC can only provide approximate information about the anti-infective effects of antimicrobial drugs, both methods are very fast, cost-effective, and suitable for screening a high number of bacterial strains with different antibiotics, and will, therefore, remain important reference values.

### 4.2. TKC

Another possible approach that has been widely used in the discussed studies are TKCs. With a previously evaluated MIC value, the TKC can be used to study antimicrobial effects, with concentrations several fold above and below the MIC, thus providing more detailed information about the time course of the antimicrobial effect in an animal or in vitro model [65]. TKC might be applied in advanced modelling, e.g., using some semi-mechanistic modelling, such as those developed by Nielsen et al. [66], and even the possible degradation of the antimicrobial drug in the test tube over 24 h can be taken into account, while this is currently neglected in MIC determinations [67].

Both a static and a dynamic approach can be taken into consideration when using TKC. With a static approach, a constant antibiotic concentration simulates a steady-state situation where the growth media is not replaced or changed. This means that all conditions for the bacteria in the culture vessel should remain unchanged throughout the observational period [68]. This could already be challenging, as not all growth media or body fluids might allow a homogenous suspension (e.g., clotting of blood components). Moreover, some components might be spent by the dividing bacteria, and antibiotics might not be stable throughout the whole experiment (e.g., tigecycline in MHB [69]).

Within TKCs, media regardless of their turbidity can be used. On the one hand, this method allows us to test multiple bacterial isolates, challenging them with different antibiotic concentrations. On the other hand, it is still an experiment in a culture vessel, a closed system, and thus bacteria might be at a certain time point hampered in their growth by limited nutrition and space, as well as toxic metabolites [68].

Like these static models, dynamic in vitro models also result in TKC. Whilst significantly more elegant, they are also much more time-consuming. Such a model, however, allows for the simulation of variable concentrations through multiple dosing by dilution or diffusion. These changing drug concentrations are obtained in systems with flowing media, which can be either open or closed systems. In a dynamic closed system, the loss of bacteria is prevented; however, as growth media dilutes the bacterial suspension, this must be taken into consideration, and might impact the results [70]. With a dynamic open system, there are two options: with the first, the bacteria float in the growth media, which results in a bacterial loss through the controlled inflow and outflow of the medium. With the second—and this is considered to be the more convenient strategy—the bacteria are retained by semipermeable membranes, which allows the bacteria to exchange with their environment (e.g., tests with a hollow fiber model [71]). A big advantage of this method is the availability of fresh broth and antibiotics throughout the experiment (constant in and outflow). Limitations within these models are that, on the one hand, the amount of adapted media or body fluid needed to proceed with the experiments (making this option impossible for most human body fluids), and on the other hand, the growth media must not clog the semipermeable membranes (e.g., erythrocytes might close the fine pores of hollow fiber systems)

In Table 4, the pros and cons for these two methods, with a focus on adapted media and body fluids as media, are listed.

## 5. Outlook

No or very scarce information is available on PD in vitro studies in breast milk, ascites, tears, saliva, gastrointestinal fluids, or even vaginal discharge or sperm. Indeed, testing in these body fluids might reveal new insights in antibiotic susceptibility testing, and might be a source for further investigations. As discussed in the prior sections of this review, it is often difficult or not feasible to obtain certain body fluids; thus, attempts have been made to produce artificial body fluids (e.g., simulated saliva, simulated lung fluid) [72]. Nevertheless, how representative the composition of these fluids compared to human body fluids is, and how feasible antibiotic susceptibility testing is in these liquids, needs further investigation. Furthermore, no standard set of experiments has been defined for antibiotic susceptibility testing in adapted media or body fluids as of now.

## 6. Summary

This review shows that the impact on bacterial killing varies between individual adapted microbiological media and direct pharmacodynamic simulations in body fluids, as well as between bacterial strains, antimicrobial agents, and the compositions of the adjuvants or the biological fluid itself. Combining standard antibiotic susceptibility testing in MHB with additional experiments in adapted MHB or in body fluids to investigate the impact of different host factors should be considered for established and novel antibiotics. Yet, no standard set of experiments has been defined as of now, and would need to be developed.

Furthermore, in vitro tests would benefit from experiments with dynamic PK/PD in vitro models, which are able to simulate the synchronous PK profiles of selected antibiotics and bacterial growth, and killing of the desired pathogen in one experimental setting. Such models would allow us to overcome the neglect of nutrient flow and the spatial limitations inherent to static in vitro studies [73,74].

This review might raise awareness of the impact of host factors in different experimental settings of in vitro antimicrobial susceptibility testing.

## 7. Methods

### Literature Search

The present review aimed to include all relevant data published between 1986 and December 2019. An efficient and targeted database search (PubMed) was achieved by using certain text keywords, such as “MIC”, “TKC”, “blood”, “serum”, “plasma”, “urine”, “CSF”, “ELF”, “bile”, “pancreatic juice”, “body fluid”, “PD”, and “in vitro”. Of the systematic literature search, 53 papers were ultimately selected that contained the information of interest. Moreover, a systematic literature search for physiologic characteristics of body fluids was done.

## Figures and Tables

**Table 1:** Compartments of the human body and their physiologic compositions.

Compartment		pH	Cell Type	Proteins	Minerals, Vitamins, Fats, and Additional Information	CO_2_	O_2_	Glucose	Ref
Blood	Hematocrit 45%	7.4	erythrocytes (4.5–5.5 million/µL)	/	/	arterial blood (32–48 mm Hg) venous blood (37–50 mm Hg)	arterial blood (83–108 mm Hg) venous blood (36–43 mm Hg)	<110 mg/dL sober, 130–140 mg/dL after carbohydrate-rich diet	[5,7,8]
			leukocytes (4000–8000/µL) → 60–70% neutrophil granulocytes, 2–3% eosinophil granulocytes, 20–30% lymphocytes, 4–5% monocytes	/	/				
			thrombocytes (150,000–350,000/µL)						
	Blood plasma 55%			90% water + 10% dissolved substances, which are composed of 70% plasma proteins/other proteins → albumin (35–40 g/L plasma), α1 globulin (3–6 g/L plasma), α2 globulin (4–9 g/L plasma), β globulin (6–11 g/L plasma), ϒ globulin (13–17 g/L plasma)	20% vitamins/minerals: urea, uric acid, creatinine, hormones, enzymes, fats (cholesterol, phospholipids, triglycerides, free fatty acids, 10% electrolytes → Na^+^, Cl^−^, Ca^2+^, K^+^, Mg^2+^, H_3_PO_4_, NaCl (0.6–0.7 g/100 mL plasma)				
Urine	Urine 0.5–2 L in 24 h	5.6–7	erythrocytes 0–3/visual field	150 mg	12–20 mg/dL urea, 0.25–0.75 g/24 h uric acid, 1.5 g creatinine, 0–0.14 g/24 h, <0.25 g/24 h glucose, 40–220 mmol/24 h Na^+^, 25–125 mmol/24 h K^+^, 2.5–7.5 mmol/24 h Ca^2+^, 110–250 mmol/24 h Cl^−^, 13–42 mmol/24 h H_3_PO_4_				[5,7,9]
			thrombocytes 0–4/visual field						
Brain, Spinal cord	CSF ~ 150mL	7.28–7.32	lymphocytes and monocytes < 5/mm^3^; no erythrocytes	lumbar CSF: 0.1––0.4g/L; ventricular CSF: 0.07–0.25 g/L	Na^+^ (135–147 mmol)/L, K^+^ (3.5–5.3 mmol)/L, Cl^−^ (95–110 mmol)/L, Ca^+^ (2.10–2.60 mmol)/L, Mg^+^ (0.8–1.1 mmol)/L, H_3_PO_4_ (0.81–1.45 mmol)/L, Fe (0.2–0.4 mmol)/L, creatinine (50–110 mmol)/L, urea (3.0–6.5 mmol)/L, lactate (1.1–2.4 mmol)/L	44–50 mmHg	40–44 mmHg	glucose (2.8–4.4 mmol)/L	[10,11]
Lung	ELF	/	Bronchial wash macrophages: 7.2 (5.2–12.3) × 10^4^ cells/mL; neutrophils: 0.7 (0.3–1.0) × 10^4^ cells/mL; lymphocytes: 0.3 (0.2–0.7) × 10^4^ cells/mL; eosinophils: 0.0 (0.0–0.1) × 10^4^ cells/mL; mast cells: 0.01 (0.00–0.02) × 10^4^ cells/mL	total protein, in mg/mL: 14.3 (11.8–24.6)	alveolar (49.2% albumin, 6.3% surfactant protein A) bronchial (63.67% albumin, 3.35% surfactant protein A)	/	/	/	[12]
			Bronchoalveolar lavage Macrophages: 13.9 (8.9–17.6) × 10^4^ cells/mL; neutrophils: 0.1 (0.0–0.2) × 10^4^ cells/mL; lymphocytes: 0.7 (0.5–1.0) × 10^4^ cells/mL; eosinophils: 0.0 (0.0–0.1) × 10^4^ cells/mL; mast cells: 0.01 (0.00–0.03) × 10^4^ cells/mL	total protein, in mg/mL: 40.4 (28.9–51.4)					
Bile	Gallbladder	6.89–7.00	/	total protein: 4.5 g/L	Carbohydrates: 2.4 g/L; chloride: 66.2 mmol/L; H_3_PO_4_: 45 mmol/L; K^+^: 12.8–24.6 mmol/L; Na^+^: 179–209 mmol/L; Ca^+^: 3.7–10.8 mmol/L; Ku: 87.5 µmol/L; zinc: 5.4 µmol/L				[13]
	Liver bile	7.15	/	total protein: 1.8–7 g/L; albumin: 634 mg/L	Cl^−:^ 105 mmol/L; H_3_PO_4_: 4.78 mmol/L; K: 4.8 mmol/L; Na^+^: 146–149 mmol/L; Ca^+^: 4 mmol/L; Fu: 15 µmol/L; Ku: 5.8–25.3 µmol/L				[13]
Pancreas	Pancreatic juice: ~17–20 mL/kg/day	7.7	/	total protein: 6.6 g/L	Cl^−^: 56 mmol/L; H_3_PO_4_: 0.8 mmol/kg; K: 7.6 mmol/L; sodium: 125 mmol/L; Ca^+^: 0.6 mmol/L; zinc: 18.5 µmol/L; chymotrypsin; trypsin’ carboxypeptidase A and B; lipase				[13]
Breast milk	Colostrum → mature milk	7.01–7.29	/	Total protein: 10.6–22.9 g/L; Whey protein (lactalbumin): 3.6–7.8 g/L	Na+: 0.172–0.501 g/L; K: 0.512–0.745 g/L; Ca+: 0.344–0.481 g/L; Mg+: 0.035–0.042 g/L; Fu: 0.50–1 mg/L; Ku: 0.51–1.34 mg/L; Zn: 1.18–5.59 mg/L; N: 324–479 mg/L; lactose: 57–71 g/L; total fat: 29.5–45.4 g/L	/	/	/	[13]

**Common bacterial infections sites with their physiologic** compositions are listed. CSF: cerebrospinal fluid; ELF: epithelial lining fluid; mmHg: abbreviation for millimeter(s) of mercury (a unit of pressure equal to the pressure that can support a column of mercury 1 mm high).

**Table 2:** Adapted media.

Adapted Media	Incorporated Bacteria	In Vitro Test	Antibiotics	Year	Ref
MHB spiked with 50% human erythrocytes	ATCC-25922 *E. coli*, ATCC-29213 *S. aureus*, ATCC-27853 *P. aeruginosa*	MIC, growth assay, TKC	Ciprofloxacin, meropenem, tigecycline	2019	[14]
MHB spiked with thrombocyte concentrates	ATCC-25922 *E. coli*, ATCC-29213 *S. aureus,* ATCC-27853 *P. aeruginosa*	MIC, growth assay, TKC	Ciprofloxacin, meropenem, tigecycline	2019	[15]
MHB spiked with 50% human serum or NaCl	209 clinical isolates of *Enterobactericae* carrying blaKPC	MIC	Cefepime–tazobactam	2017	[16]
MHB with 12% albumin pH 6 and pH 7.4 at 32 °C, 37 °C, and 42 °C	20 clinical isolates of *S. aureus;* ATCC-29213 *S. aureus*	MIC, growth assay, TKC	Telavancin, vancomycin, teicoplanin	2018	[17]
MHB with 4%, 8%, 12%, and 16% human albumin MHB with 20%, 50%, and 70% human serum	ATCC-29213 *S. aureus*	MIC, growth assay, TKC	Clindamycin	2010	[18]
Pure MHB and 100% serum MHB with 4%, 8%, 12%, and 16% human albumin MHB with 20% and 70% human serum	ATCC-29213 *S. aureus*, ATCC-27853 *P. aeruginosa*	Ultrafiltration, MIC, growth assay, TKC,	Moxifloxacin, trovafloxacin	2007	[19]
Pure MHB and MHB with 50% plasma	40 methicillin-susceptible *S. aureus* (MSSA); 38 methicillin-resistant *S. aureus* (MRSA)	MIC	Vancomycin, fusidic-acid, teicoplanin, iclaprim	2007	[20]
MHB with 40 mg/L human albumin	ATCC-29213 *S. aureus*	MIC, growth assay, TKC,	Ampicillin, fosfomycinosfomycin, oxacillin, moxifloxacin	2004	[21]
Pure MHB and MHB with 65% defibrinated human blood	17 *E. faecium* clinical isolates, *E. faecalis ATCC29212*, *S. aureus ATCC25923*	MIC, MBC, TKC	Ampicillin, ciprofloxacin, co-trimoxazole, doxycycline, fusidic-acid, imipenem, gentamicin, mupirocin, novobiocin, rifampin, streptomycin, taurolidine, teicoplanin, trimethoprim-sulfamethoxazole, vancomycin	1993	[22]
Pure MHB and MHB with 65% defibrinated human blood	Six MDR *S. aureus* isolates, *S. aureus* ATCC25923	MIC, MBC	Amikacin, cefamandole, chloramphenicol, ciprofloxacin, clindamycin, coumermycin, teicoplanin, fusidic acid, gentamycin, imipenem, netilmicin, novobiocin, ofloxacin, oxacillin, rifampicin, tobramycin, trimethoprim-sulfamethoxazole, vancomycin	1991	[23]
MHB and MHB with 90%, 80%, and 50% urine	16 urogenic clinical isolates of *Enterobacteriaceae*	MIC	delafloxacin, ciprofloxacin	2016	[24]

In column 1, the adapted growth medium is shown. Column 2 depicts the pathogens tested. and columns 3 and 4 show the in vitro methods used and the antibiotics tested, respectively. MHB: Mueller–Hinton broth, MIC: minimal inhibitory concentration, TKC: time kill curve, MBC: minimum bactericidal concentration.

**Table 3:** Body fluids as media.

Body Fluid	Incorporated Bacteria	In Vitro Test	Antibiotics	Year	Ref
Pooled human urine, MHB	Clinical isolates of *E. coli*, *K. pneumoniae*, and *P. mirabilis*	MIC, TKC	Ceftolozane–tazobactam, meropenem	2019	[25]
Pooled human bile fluid	ATCC-25922 *E. coli,* ATCC-29212 *E. faecalis*	MIC, TKC	Linezolid, tigecycline, meropenem, ciprofloxacin	2017	[26]
MHB, urine, artificial urine medium	*E. coli* MG1655 wild-type (DA5438); cysB deletion mutant MG1655 (DA28439); MecR clinical *E. coli* UTI isolates DA14719, DA24682, and DA24686	MIC (test strips and Etest), growth measurement (Bioscreen C Analyser)	Mecillinam, meropenem, ampicillin, cefotaxime	2017	[27]
MHB, urine	Six urogenic clinical isolates: *E. coli, S. saprophyticus, K. pneumoniae, P. mirabilis, E. faecalis,* and *S. epidermidis*	MIC, disc-diffusion assay	Ciprofloxacin, ofloxacin, levofloxacin, gentamicin, tobramycin, erythromycin, azithromycin, trim/Sulfa, trimethoprim, tetracycline, doxycycline, cefotaxime, cephalothin, cefazolin, ceftazidime, ampicillin, piperacillin, nitrofurantoin	2014	[28]
MHB, urine	ATCC-25922 *E. coli*, ATCC-29213 *S. aureus,* ATCC-700324 *K. oxytoca,* ATCC-14153 *P. mirabilis,* ATCC-29212 *E. faecalis*	MIC, growth assay, TKC	Trimethoprim, fosfomycin, colistin, amikacin, ertapenem	2012	[29]
MHB, pooled human CSF in 5% CO_2_, artificial substitute CSF, 0.5 g/L human albumin, sodium OH (0.064 M solution)	ATCC-29213 *S. aureus,* ATCC-12228 *S. epidermidis*	MIC, growth assay, TKC	Linezolid	2012	[30]
MHB, urine	ATCC-25922 *E. coli*, ATCC-700324 *K. oxycota*	MIC, growth assay, TKC	Ciprofloxacin, levofloxacin, moxifloxacin	2010	[31]
MHB, pooled human CSF in ambient air, pooled human CSF in 5% CO_2_	ATCC-29213 *S. aureus*, clinical isolate of *S. aureus* (MIC 16mg/L)	TKC	Fosfomycin	2009	[32]
Reference MHB, pooled human CSF in ambient air, pooled human CSF in 5% CO_2_, pooled human CSF in 5% CO_2_	ATCC-29213 *S. aureus*	TKC	Cefepime, rifampicin	2008	[33]

In column one, the tested body fluid is shown. Column 2 depicts the pathogens tested, and columns 3 and 4 show the in vitro methods used and the antibiotics tested, respectively. MHB: Mueller–Hinton broth, MIC: minimal inhibitory concentration, TKC: time kill curve, MBC: minimum bactericidal concentration, CSF: cerebrospinal fluid.

**Table 4:** Pros and cons for MIC and TKCs, with adapted media and body fluids as media.

	Advantages	Limitations
MIC/MBC	Easy and quick to perform	Static approach
	Screening of a high number of isolates	Problems with turbidity in MIC testing (i.e., with blood, urine, surfactant)
	Widely used: EUCAST * and CLSI **	Two-fold dilution steps might not detect small changes in the efficacy of the tested concentration
	Guide values for further testing	Kinetics of bacterial killing are not recorded
		MIC is determined by visible growth (10^7^ cells/mL); low growth is not considered
		Difficulties with swarming bacteria or bacteria that produce CO_2_ (e.g., *Proteus mirabilis* in urine)
		Instability of antibiotics in MHB or adapted MHB
		Components of the culture media might be spent by dividing bacteria
TKC	Kinetics of bacterial killing can be observed and time-CFU/mL *** curves can be produced	Labor-intensive and time consuming—not suitable for screening
	A coherent system with killing curves and growth assays at the same time	Amount of the volume of adapted media or body fluid is rather high
	Possibility of mimicking multiple dosing and **** T > MIC	Maintaining a homogenous suspension might be difficult
	Time points can be chosen individually, and CFU/mL *** can be evaluated at multiple time points	Instability of antibiotics in MHB or adapted MHB
	No problems with the turbidity of media	Components of the culture media might be spent by dividing bacteria
	Fresh broth and antibiotic in HF systems	

Table 4 summarizes the advantages and limitations of the minimal inhibitory concentration (MIC), the minimum bactericidal concentration (MBC) and time kill curves (TKCs). * EUCAST: European Committee on Antimicrobial Susceptibility Testing; ** CLSI: Clinical and Laboratory Standards Institute; *** CFU: colony-forming unit; HF: hollow fiber; **** Time over the Minimal Inhibitory Concentration (T > MIC).

## Supplementary Materials

The following are available online at https://www.mdpi.com/1999-4923/12/8/773/s1, Table S1: Antibiotic susceptibility testing in adjusted growth media and in body fluids, and their impact on antibiotic activity.
