# Supplementary material for: Use of Supplemented or Human Material to Simulate PD Behavior of Antibiotics at the Target Site In Vitro
Source: Pharmaceutics. 2020 Aug 14;12(8):773. doi: 10.3390/pharmaceutics12080773 (PMC7464672; doi:10.3390/pharmaceutics12080773)
Supplement: Supplementary file 1 [file pharmaceutics-12-00773-s001.pdf]

Supplementary Materials: Use of Supplemented or Human Material to Simulate PD Behavior of Antibiotics at the Target Site In Vitro

Alina Nussbaumer-Pröll [1] and Markus Zeitlinger [2,*]

[1] Department of Clinical Pharmacology, Medical University of Vienna, 1090, Vienna, Austria; alina.nussbaumer-proell@meduniwien.ac.at
[2] Department of Clinical Pharmacology, Medical University of Vienna, 1090, Vienna, Austria
* Correspondence: markus.zeitlinger@meduniwien.ac.at

Table S1. Antibiotic susceptibility testing in adjusted growth media and in body fluids and their impact on antibiotic activity. .

Adjusted MHB/Body Fluid	Used Antibiotic	Bacterial strain/s	Antibiotic activity	Reference
**Serum+MHB**	ertapenem	*E. cloacae* and *S. aureus* isolates	↓	[1]
**Serum+MHB**	cefepime-tazobactam	KPC-producing *Enterobacteriaceae*	↑	[2]
**Serum+MHB**	ampicillin	*Enterococci* isolates	↓	[3]
**Serum+MHB**	telavancin	Glycopeptide non-susceptible *Staphylococci*, Vancomycin resistant *S. aureus* strains	↓	[4]
**Serum+Todd-Hewitt-Broth**	ciprofloxacin, levofloxacin, trovafloxacin, moxifloxacin, clinafloxacin, gemifloxacin	*S. pneumoniae* isolates	moxifloxacin ↔,All other tested AB + serum ↓	[5]
**Serum+MHB, Albumin+MHB**	daptomycin, telavancin, teicoplanin, vancomycin	*S. aureus* isolates	All tested AB + serum ↓<br>vancomycin + albumin ↔<br>daptomycin + albumin ↓	[6]
**Serum+MHB, Albumin+MHB**	telavancin	*S. aureus* (methicillin resistant and methicillin susceptible)	↔	[7]
**Serum+MHB, Albumin+MHB**	clindamycin	S. aureus ATCC-29213	↓	[8]
**Serum+MHB, Albumin+MHB**	cefditoren	penicillin-resistant *S. pneumoniae* isolates	cefditoren+albumin ↓<br>cefditoren+90% human serum ↔	[9]
**Pure serum, pure MHB (control), albumin+MHB, serum+MHB**	moxifloxacin, trovafloxacin	*S. aureus* ATCC-29213, *P. aeruginosa* ATCC-27853	↓	[10]
**Albumin+MHB**	moxifloxacin, ampicillin, oxacillin, fosfomycin (negative control)	*S. aureus* ATCC-29213	fosfomycin ↔,<br>All others ↓	[11]
**Albumin+MHB**	daptomycin	MRSA, vancomycin resistant *E. faecium*	↔	[12]
**Plasma+MHB**	iclaprim	MRSA, MSSA isolates	↔	[13]

**Defibrinated blood+MHB**	rifampin, rifampin + fusidic acid,<br>rifampin + co-trimoxazole,<br>mupirocin,<br>taurolidine	17 *E. faecium* clinical isolates	rifampin, rifampin + fusidic acid ↔,rifampin + co-trimoxazole ↔,<br>mupirocin ↓<br>taurolidine ↓	[14]
**Defibrinated blood+MHB**	teicoplanin,<br>vancomycin,<br>teicoplanin + fusidic acid,<br>teicoplanin + fusidic acid,<br>teicoplanin + other drug combinations	MDR *S. aureus* isolates	teicoplanin ↓,<br>vancomycin ↓,<br>teicoplanin + fusidic acid ↓,<br>teicoplanin + fusidic acid ↓,<br>teicoplanin + other drug combinations ↓	[15]
**Defibrinated blood+MHB**	ampicillin, imipenem, mezlocillin, ofloxacin, piperacillin, vancomycin	*S. faecalis* isolates	↔	[16]
**Defibrinated blood+MHB**	cefoperazone, gentamicin, netilmicin, aztreonam, ceftazidime, ciprofloxacin, fleroxacin, imipenem, tobramycin, azlocillin, ceftazidime, norfloxacin, ofloxacin, piperacillin, ticarcillin,	*P. aeruginosa* isolates	cefoperazone ↔,<br>gentamicin↔, netilmicin↔,<br>All further drug combinations ↑,	[17]
**Defibrinated blood+MHB**	cefepime, ceftazidime, rifampin, and timentin, ofloxacin, cotrimoxazole, polymyxin B,	*Stenotrophomonas* (*Xanthomonas*) *maltophilia* isolates	Various combinations of tested AB ↑	[18]
**Defibrinated blood+MHB**	imipenem, polymyxin B, amikacin, ceftazidime, ciprofloxacin,	*Acinetobacter calcoaceticus*	Various combinations of tested AB ↑	[19]
**MHB+50% erythrocytes**	ciprofloxacin, meropenem, tigecycline	*E. coli ATCC-25922, S. aureus ATCC-29213, P. aeruginosa ATCC-27853*	↓	[20]

**MHB+thrombocyte concentrates**	ciprofloxacin, meropenem, tigecycline	*E. coli ATCC-25922, S. aureus ATCC-29213, P. aeruginosa ATCC-27853*	Dependent on thrombocyte concentrate	[21]
**Urine+MHB+ different pH settings**	ciprofloxacin, norfloxacin, ofloxacin	*E. coli, S. aureus, P. aeruginosa* isolates	Acidic pH ↓	[22]
**Urine+MHB+ different pH settings**	ciprofloxacin, levofloxacin, moxifloxacin	*E. coli* ATCC-25922, *K. oxycota* ATCC-29213	Acidic pH ↓	[23]
**Urine+MHB+ different pH settings**	trimethoprim, fosfomycin, amikacin, colistin, ertapenem	*E. coli* ATCC-25922, *K. oxycota* ATCC-29213, *P. mirabilis* ATCC-14153, *E. faecalis* ATCC-29212	Acidic pH ↓	[24]
**Urine+MHB+ different pH settings**	24 widely used antimicrobial agents	*E. coli, K. pneumoniae, P. mirabilis, E. faecalis, Staphylococcus saprophyticus, Staphylococcus epidermidis*	Activity varied between antibiotics tested (acidic pH↓ and acidic pH↑)	[25]
**CSF + MHB**	cefepime, rifampicin	*S. aureus* ATCC-29213	cefepime ↑, rifampicin ↓	[26]
**CSF+MHB**	linezolid	*S. aureus* ATCC-29213, *S. epidermidis* ATCC-12228	linezolid ↑	[27]
**CSF+MHB**	fosfomycin	*S. aureus* ATCC-29213	fosfomycin ↓	[28]
**Bile**	linezolid, tigecycline, meropenem, ciprofloxacin	*E. faecalis* ATCC-29212, *E. coli* ATCC-25922	↓	[29]
**Cations Ca^+ and Mg^++MHB**	colistin	*P. aeruginosa, A. baumannii, E. coli*	Increase in AB activity against *P. aeruginosa* and *A. baumannii*, Decrease of AB activity against *E. coli*	[30]

**MHB+Lung Surfactant (bovine lung surfactant)**	amoxicillin, ceftazidime, tobramycin,	*P. aeruginosa ATCC-27853, S. aureus ATCC-25923, S. pneumoniae ATCC-6301, K. pneumoniae ATCC-43816*	ceftazidime ↔, amoxicillin ↔, tobramycin ↓,	[31]
**MHB+Lung Surfactant (Survanta)**	daptomycin	*S. aureus* ATCC-29213	↓	[32]
**MHB+Lung Surfactant (Curosurf)**	daptomycin, linezolid, doripenem, tigecycline moxifloxacin,	*S. aureus* ATCC-29213	↓	[33]
**MHB+Lung Surfactant (Surventa)**	telavancin, daptomycin, ceftriaxone, vancomycin,	MRSA	telavancin ↔, daptomycin ↓, ceftriaxone ↔, vancomycin ↔	[34]
**MHB+Lung Surfactant (Surventa)**	Expanded spectrum lipopeptide MX-2401 (semisynthetic analogue to amphomycin), daptomycin, vancomycin	*S. aureus* (MRSA, MSSA, etc.) *S. pneumoniae*	daptomycin ↓, vancomycin ↔, MX2401 ↔,	[35]

Table S1: This table summarizes an excerpt of studies dealing with antibiotic susceptibility testing with adjusted growth media and body fluids. This table is organized in 5 columns; column 1 shows the adapted growth media or body fluid, column 2 describes the tested antibiotics, column 3 displays the tested bacterial strains, column 4 describes the impact on antibiotic activity of the adjuvants or the body fluid (↓ impaired, ↑ enhanced and ↔ equal activity). Column 5 lists the references.

References

1. Nix, D.E.; Matthias, K.R.; Ferguson, E.C. Effect of ertapenem protein binding on killing of bacteria. Antimicrob. *Agents Chemother.* **2004**, *48*, 3419–3424.
2. Castanheira, M.; Duncan, L.R.; Rhomberg, P.R.; Sader, H.S. Enhanced activity of cefepime–tazobactam (WCK 4282) against KPC-producing Enterobacteriaceae when tested in media supplemented with human serum or sodium chloride. *Diagn. Microbiol. Infect. Dis.* **2017**, *89*, 305–309.
3. Gilbert, D.N.; Eubanks, N. Effect of pH and Human Serum on the Susceptibility of Group D Streptococci (Enterococci) to Ampicillin In Vitro. Antimicrob. *J. Antimicrob. Chemother.* **1975**, *7*, 387–395.
4. Leuthner, K.D.; Cheung, C.M.; Rybak, M.J. Comparative activity of the new lipoglycopeptide telavancin in the presence and absence of serum against 50 glycopeptide non-susceptible staphylococci and three vancomycin-resistant Staphylococcus aureus. *J. Antimicrob. Chemother.* **2006**, *58*, 338–343.
5. Balcabao, I.P. Influence of the decrease in ciprofloxacin susceptibility and the presence of human serum on the in vitro susceptibility of Streptococcus pneumoniae to five new quinolones. *J. Antimicrob. Chemother.* **2001**, *48*, 907–909.
6. Tsuji, B.T.; Leonard. S.N.; Rhomberg, P.R.; Jones, R.N.; Rybak, M.J. Evaluation of daptomycin, telavancin, teicoplanin, and vancomycin activity in the presence of albumin or serum. *Diagn. Microbiol. Infect. Dis.* **2008**, *60*, 441–444.
7. Odenholt, I.; Löwdin, E.; Cars, O. Pharmacodynamic effects of telavancin against methicillin-resistant and methicillin-susceptible Staphylococcus aureus strains in the presence of human albumin or serum and in an in vitro kinetic model. Antimicrob. *Agents Chemother.* **2007**, *51*, 3311–3316.
8. Burian, A.; Wagner, C.; Stanek, J.; Manafi, M.; Böhmdorfer, M.; Jäger, W.; Zeitlinger, M. Plasma protein binding may reduce antimicrobial activity by preventing intra-bacterial uptake of antibiotics, for example clindamycin. *J. Antimicrob. Chemother.* **2011**, *66*, 134–137.
9. Sevillano, D.; Giménez MJ, Alou, L.; Aguilar, L.; Cafini, F.; Torrico, M.; González, N.; Echeverría, O.; Coronel, P.; Prieto, J. Effects of human albumin and serum on the in vitro bactericidal activity of cefditoren against penicillin-resistant Streptococcus pneumoniae. *J. Antimicrob. Chemother.* **2007**, *60*, 156–158.
10. Zeitlinger, M.; Sauermann, R.; Fille, M.; Hausdorfer, J.; Leitner, I.; Müller, M. Plasma protein binding of fluoroquinolones affects antimicrobial activity. *J. Antimicrob. Chemother.* **2008**, *61*, 561–567.
11. Zeitlinger, M.A.; Sauermann, R.; Traunmüller, F.; Georgopoulos, A.; Müller, M.; Joukhadar, C. Impact of plasma protein binding on antimicrobial activity using time-killing curves. *J. Antimicrob. Chemother.* **2004**, *54*, 876–880.
12. Cha, R.; Rybak, M.J. Influence of protein binding under controlled conditions on the bactericidal activity of daptomycin in an in vitro pharmacodynamic model. *J. Antimicrob. Chemother.* **2004**, *54*, 259–262.
13. Laue, H.; Valensise, T.; Seguin, A.; Hawser, S.; Lociuro, S.; Islam, K. Effect of human plasma on the antimicrobial activity of iclaprim in vitro. *J. Antimicrob. Chemother.* **2007**, *60*, 1388–1390.
14. Walter, H.; Leonhard, B.; Bauer, D. Enterococcus faecium: In vitro Activity of Antimicrobial Drugs, Singly and Combined, with and without Defibrinated Human Blood, against Multiple-Antibiotic-Resistant Strains. *Chemotherapy* **1993**, *39*, 254–264.
15. Traub, W.H.; Spohr, M.; Bauer, D. Teicoplanin combined with various antibiotics and human blood against a multiple-drug-resistant strain of staphylococcus aureus. *Chemotherapy* **1991**, *37*, 186–195.
16. Traub, W.H.; Sphor, M.; Bauer, D. Streptococcus faecalis: In vitro Susceptibility to Antimicrobial Drugs, Single and Combined, with and without Defibrinated Human Blood. *Chemotherapy* **1986**, *32*, 270–285.
17. Traub, W.H.; Spohr, M.; Bauer, D. Pseudomonas aeruginosa: In vitro susceptibility to antimicrobial drugs, single and combined, with and without defibrinated human blood. *Chemotherapy* **1988**, *34*, 284–297.
18. Traub, W.; Leonhard, B.; Bauer, D. Stenotrophomonas (Xanthomonas) maltophilia: In vitro susceptibility to selected antimicrobial drugs, single and combined, with and without defibrinated human blood. *Chemotherapy* **1998**, *44*, 293–304.
19. Traub, W.H.; Spohr, M.; Bauer, D. Susceptibility of Acinetobacter calcoaceticus to antimicrobial drugs, alone and combined, with and without defibrinated human blood. *Chemotherapy* **1989**, *35*, 95–104.
20. Nussbaumer-Pröll, A.K.; Knotzer, S.; Eberl, S.; Reiter, B.; Stimpfl, T.; Jäger, W.; Poschner, S.; Zeitlinger, M. Impact of erythrocytes on bacterial growth and antimicrobial activity of selected antibiotics. *Eur. J. Clin. Microbiol. Infect. Dis.* **2019**, *38*, 485–495.

21. Nussbaumer-Pröll, A.K.; Eberl, S.; Reiter, B.; Stimpfl, T.; Jäger, W.; Poschner, S.; Zeitlinger, M. Impact of erythrocytes on bacterial growth and antimicrobial activity of selected antibiotics. *Eur. J. Clin. Microbiol. Infect. Dis.* **2020**, *39*, 593–597.
22. Zeiler, H.-J. Influence of pH and human urine on the antibacterial activity of ciprofloxacin, norfloxacin and ofloxacin. *Drugs Exp. Clin. Res.* **1985**, *11*, 335–338.
23. Erdogan-Yildirim, Z.; Burian, A.; Manafi, M.; Zeitlinger, M. Impact of pH on bacterial growth and activity of recent fluoroquinolones in pooled urine. *Res. Microbiol.* **2011**, *162*, 249–252.
24. Burian, A.; Erdogan, Z.; Jandrisits, C.; Zeitlinger, M. Impact of pH on activity of trimethoprim, fosfomycin, amikacin, colistin and ertapenem in human urine. *Pharmacology* **2012**, *90*, 281–287.
25. Yang, L.; Wang, K.; Li, H.; Densted JD.; Cadieux, P.A. Re: The influence of urinary pH on antibiotic efficacy against bacterial uropathogens. *J. Urol.* **2015**, *193*, 151.
26. Sauermann, R.; Schwameis, R.; Fille, M.; Camuz Ligios, M.L.; Zeitlinger, M. Antimicrobial activity of cefepime and rifampicin in cerebrospinal fluid in vitro. *J. Antimicrob. Chemother.* **2008**, *62*, 1057–1060.
27. Schwameis, R.; Fille, M.; Manafi, M.; Zeitlinger, M.; Sauermann, R. Enhanced activity of linezolid against Staphylococcus aureus in cerebrospinal fluid. *Res. Microbiol.* **2012**, *163*, 157–160.
28. Sauermann, R.; Schwameis, R.; Fille, M.; Camuz ligios, M.L.; Zeitlinger, M. Cerebrospinal fluid impairs antimicrobial activity of fosfomycin in vitro. *J. Antimicrob. Chemother.* **2009**, *64*, 821–823.
29. Wulkersdorfer, B.; Jaros, D.; Poschner, S.; Jäger, W.; Cosentini, E.; Zeitlinger, M.; Schwameis, R. Human Bile Reduces Antimicrobial Activity of Selected Antibiotics against Enterococcus faecalis and Escherichia coli In Vitro. Antimicrob. *Agents Chemother.* **2017**, *61*, 1–9.
30. Matzneller, P.; Strommer, S.; Österreicher, Z.; Mitteregger, D.; Zeitlinger, M. Target site antimicrobial activity of colistin might be misestimated if tested in conventional growth media. *Eur. J. Clin. Microbiol. Infect. Dis.* **2015**, *34*, 1989–1994.
31. Van't Veen, A.; Mouton, J.W.; Gommers, D.; Kluytmans, J.A.N.A.J.W.; Dekkers, P. Influence of Pulmonary Surfactant on In Vitro Bactericidal Activities of Amoxicillin, Ceftazidime, and Tobramycin. *Antimicrob. Agents Chemother.* **1995**, *39*, 329–333.
32. Silverman, J.A.; Mortin, L.I.; Vanpraagh, A.D.G.; Li, T.; Alder, J. Inhibition of Daptomycin by Pulmonary Surfactant: In Vitro Modeling and Clinical Impact. *J. Infect. Dis.* **2005**, *191*, 2149–2152.
33. Schwameis, R.; Erdogan-yildirim, Z.; Manafi, M.; Zeitlinger, M.A.; Strommer, S.; Sauermann, R. Effect of Pulmonary Surfactant on Antimicrobial Activity In Vitro. Antimicrob. *Agents Chemother.* **2013**, *57*, 5151–5154.
34. Gotfried, M.H.; Shaw, J.; Benton, B.M.; Krause, K.M.; Goldberg, M.R.; Kitt, M.M.; Barriere, S.L. Intrapulmonary Distribution of Intravenous Telavancin in Healthy Subjects and Effect of Pulmonary Surfactant on In Vitro Activities of Telavancin and Other Antibiotics. Antimicrob. *Agents Chemother.* **2008**, *52*, 92–97.
35. Dugourd, D.; Yang, H.; Elliott, M.; Siu, R.; Clement, J.J.; Straus, S.K.; Hancock, R.E.W.; Rubinchik, E. Antimicrobial Properties of MX-2401, an Expanded-Spectrum Lipopeptide Active in the Presence of Lung Surfactant. Antimicrob. *Agents Chemother.* **2011**, *55*, 3720–3728.

© 2020 by the authors. Submitted for possible open access publication under the terms and conditions of the Creative Commons Attribution (CC BY) license (http://creativecommons.org/licenses/by/4.0/).
